# Is Tumor-Free Distance an Independent Prognostic Factor for Early-Stage Endometrioid Endometrial Cancer?

**DOI:** 10.1155/2020/2934291

**Published:** 2020-04-14

**Authors:** Tufan Oge, Duygu Kavak Comert, Yusuf Cakmak, Deniz Arık

**Affiliations:** ^1^Department of Gynecologic Oncology, Eskişehir Osmangazi University School of Medicine, Eskişehir, Turkey; ^2^Department of Pathology, Eskişehir Osmangazi University School of Medicine, Eskişehir, Turkey

## Abstract

There are many studies assessing the importance of myometrial invasion using a cut-off limit as 50% of myometrial invasion for endometrial cancer, and there are a limited number of studies evaluating tumor-free distance to the serosa. To evaluate the prognostic performance of tumor-free distance and percentage of myometrial invasion in patients with stage IB endometrioid endometrial cancer, we retrospectively evaluated 133 patients diagnosed and treated as stage IB endometrioid endometrial cancer. Tumor-free distance was assessed, and recurrence and recurrence-free survival were analyzed. Nine patients had recurrent disease (6.8%). Recurrence-free survival was 200 months. Two patients died because of malignancy. In the Cox regression model according to tumor-free distance, depth of invasion, and percentage of myometrial invasion, it was seen that none of these parameters were significant to predict the recurrence (*p* > 0.05). In conclusion, tumor-free distance is not an independent prognostic factor for patients with stage IB endometrioid endometrial cancer.

## 1. Introduction

Endometrial cancer is surgically staged and the FIGO (International Federation of Gynecology and Obstetrics) 2009 surgical staging system is being used for this purpose. The difference between stage IA and stage IB lies in the existence of less than half myometrial invasion or equal or more than half of the myometrium [1]. Intraoperative frozen section is used to determine the myometrial invasion depth and that is one of the criteria to extend the surgery [2]. While the surgery is the primary treatment of endometrial cancer, patients who have risk of recurrence must be referred to adjuvant treatment like pelvic radiotherapy or vaginal brachytherapy [3]. Adjuvant therapy recommended by ESGO and NCCN for endometrial cancer shows similarities. In 2016, patients were divided into risk groups by ESMO-ESGO-ESTRO Consensus Conference on Endometrial Cancer. Patients with myometrial invasion greater than half of the myometrium (stage IB) are classified in the intermediate, high-intermediate, and high-risk groups according to grade and lymphovascular space invasion; each group refers to a specific adjuvant treatment in accordance with the risk of recurrence [4].

As FIGO stage I disease is the most frequently seen endometrial cancer [5], myometrial invasion depth is one of the most important indicators that clinicians and pathologists investigate. Although there are many studies assessing the importance of myometrial invasion (MI) using a cut-off limit as 50% of MI [6–9], there are limited number of studies evaluating tumor-free distance (TFD) to the serosa [5, 10, 11]. Moreover, most of them evaluated TFD for prediction for lymph node metastasis but the long-term follow-up is taken into consideration in only few studies [12–15]. However, all these studies compare all stages of endometrial cancer patients and we argue that evaluating all the stages at the same time can generate conflicting findings. From this point of view, we hypothesize that TFD to the serosa may be an important prognostic factor regardless of lymph node and cervical involvement. To test this hypothesis, we evaluated prognostic performance of TFD, %MI, and depth of invasion (DOI) in patients diagnosed as stage IB endometrioid type endometrial cancer.

## 2. Materials and Methods

We retrospectively evaluate endometrial cancer patients treated at our institution between 2004 and 2012. Patients who were diagnosed as endometrioid endometrial cancer and staged surgically were involved the studys. Our inclusion criteria were diagnosis of stage IB endometrial cancer according to the FIGO 2009 staging system and a correct follow-up. Our exclusion criteria were nonendometrioid histology, stages other than IB, not performed surgery, not performed lymphadenectomy, incomplete records and pathologic information, and having no follow-up visits. A total of 612 endometrial cancer patients were found from the hospital database system, and 133 of these patients met the inclusion criteria.

For surgical staging exploration of pelvis and abdomen, peritoneal washings for cytology, total abdominal hysterectomy, bilateral salpingo-oophorectomy, and pelvic and para-aortic lymphadenectomy were performed. Sixteen patients were inadvisable for full lymphadenectomy because of their medical conditions. These patients were excluded from the research sample.

Patients' age at diagnosis, comorbidities, menopausal status, histologic types, grades, myometrial thickness (MT), depth of invasion (DOI), tumor-free distance (TFD), tumor diameter (TD), percentage of myometrial invasion (%MI), stages (according to FIGO 2009), existence of lymphovascular space invasion, operations data, adjuvant therapy, follow-up records, recurrence and survival data, pathology and operation records, and patients health records were obtained from the hospital database system. MT was accepted as the distance between the endometrial-myometrial junction and the serosa. DOI was measured from the endometrial-myometrial junction to the deepest point of the myometrial invasion. The percentage of myometrial invasion was calculated as the DOI divided by the myometrial thickness and multiplied by 100. TFD was defined as the distance between the uterine serosa and the deepest point of the myometrial invasion. TD was obtained from the pathology records. TD was noted in three measurements and the maximum dimension was used. The patients were followed up every 3 months for the first 2 years, every 6 months for the next 3 years, and just once in a year afterwards. Biopsy or imaging studies were used to detect the recurrence cases.

Univariate and multivariate models were used to predict the recurrence. Kaplan-Meier survival analysis was performed for recurrence-free survival. Recurrence-free survival was considered as the time between surgery and the date of recurrence. Statistical analysis was performed using SPSS 24 (SPSS Inc., Chicago, IL, USA). *p* value of <0.05 was considered statistically significant.

The local institutional ethics committee approved the study protocol.

## 3. Results

**Figure 1:**
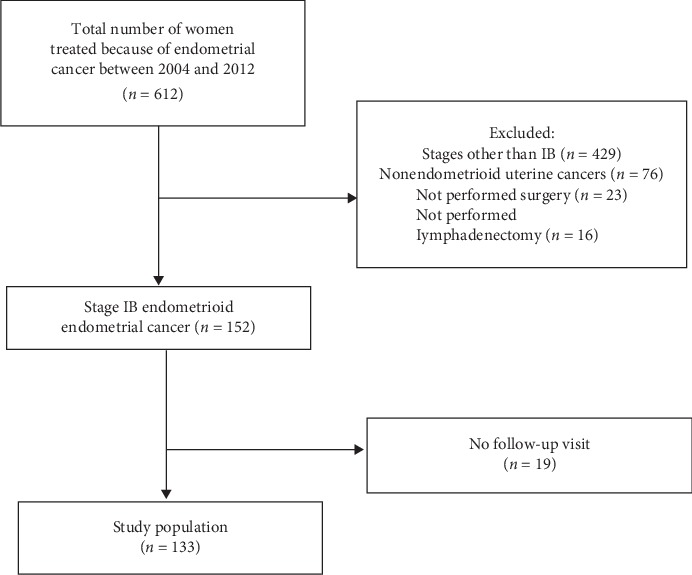
Study population.

A total of 612 patients were treated because of endometrial cancer at our institution between 2004 and 2012, and 479 patients were excluded from the study (nonendometrioid type endometrial cancer, stages other than IB, not performed surgery, not performed lymphadenectomy, incomplete records and pathologic information, and having no follow-up visits). One hundred thirty-three patients were included in the study (Figure 1).

**Figure 2:**
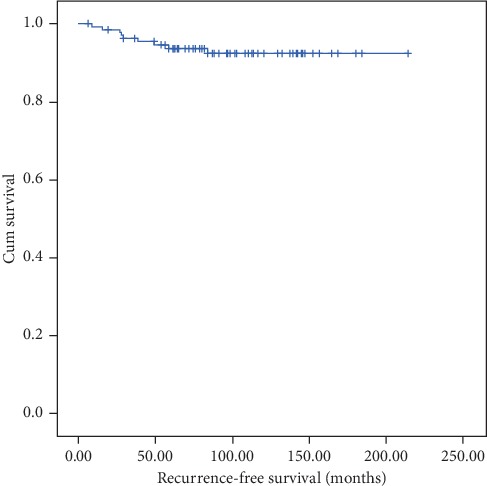
Kaplan-Meier curve shows the recurrence-free survival.

The mean age at diagnosis was 61.5 years. The mean number of lymph nodes extracted from pelvic and para-aortic regions was 38. Of these 133 patients, the mean MT was 20 mm, DOI was 14 mm, TFD was 6 mm, and TD was 35 mm. There was no difference between TFD of recurrent disease and nonrecurrent disease (*p* > 0.05). The mean TFD in patients with recurrent disease was 5 mm and in patients without recurrence was 6.2 mm. Lymphovascular space invasion (LVSI) was identified in 66 patients (49.6%). Only one patient had positive cytology (0.8%). Sixty-one patients were in the intermediate-risk group, forty-two patients were in the high-intermediate risk group, and thirty patients were in the high-risk group according to ESMO-ESGO-ESTRO Consensus Conference on Endometrial Cancers classification of risk groups (Table 1). All patients were referred to adjuvant treatment. External beam radiotherapy was performed in 15 patients, brachytherapy was performed in 98 patients, and external beam radiotherapy and brachytherapy were performed 20 of these patients. Average follow-up time was 95 months. Nine patients had recurrent disease (6.8%). While seven patients had local recurrence, two patients had systemic recurrence (lung and vertebra). The mean recurrence time was 37.6 months, and recurrence-free survival was 200 months in Kaplan-Meier analysis (Figure 2). Two patients died because of malignancy (Table 2).

When patients were analyzed with the Cox regression model according to TFD, DOI, and %MI, it was seen that none of these parameters were significant to predict the recurrence (*p* > 0.05) in both univariate and multivariate analyses (Tables 3-4).

## 4. Discussion

Our study demonstrates that the TFD, DOI, and %MI do not change the prognosis of the patients and patients' recurrence rates in stage IB endometrioid endometrial cancer.

In this study, we evaluated only patients who have stage IB endometrial cancer and excluded other types of uterine cancers and stages other than IB. Because earlier studies compare all stages of endometrial cancer patients, we want to assess if there is an importance of TFD, %MI, and DOI to the recurrence and prognosis regardless of lymph node and cervical involvement in patients with endometrioid endometrial cancer.

DOI has been used as a prognostic factor for many years. In 1984, it was shown that deep myometrial invasion was a risk factor for extra uterine disease [16]. However, sometimes it is difficult to determine DOI because of adenomyosis, leiomyoma, and irregular endomyometrial junction [12]. From this point of view, some studies started to investigate the role of TFD as a prognostic factor. One of the earliest study in 1994, Kaku et al. assessed three different methods of measuring myometrial invasion in stage I and II endometrial carcinoma patients. They found that the distance between the uterine serosa and the deepest point of the myometrial invasion was correlated with the survival. Even though this was one of the preliminary important studies to evaluate TFD as a prognostic factor, the study population was small (*n* = 88) [17]. A retrospective study, evaluating if TFD is more predictive than DOI in surgically staged endometrial cancer patients, suggested that TFD but not DOI could be used to predict the recurrence. They stated that the ROC curve demonstrated TFD of 1 cm maximized the sensitivity and specificity of disease recurrence. In this study, complete surgically staged patients were included, and nonepithelial histology subtypes were excluded [14]. In 2009, from the same center, a prospective study evaluated the value of TFD in surgically staged endometrial cancer patients. The study demonstrated that TFD is one of the important prognostic factors in endometrial cancer and it leads to less confusion than the measurement of DOI [12]. In 2010, Chennakesavan et al. compared TFD, DOI, and %MI to predict lymph node metastasis in 338 surgically staged endometrial cancer patients. The study noted that the DOI could predict the nodal involvement, but the TFD could not [11]. In 2012, the prognostic performance of TFD, DOI, and %MI were investigated in 288 stage I–III endometrioid endometrial cancer patients by Chattopadhyay et al. This retrospective study showed that TFD was an independent predictor of death from disease, recurrence, and lymph node involvement and they proposed a TFD cut-off value of 1.75 mm [15]. In 2013, another retrospective study tried to show the prognostic value of DOI, TFD, and %MI. They identified that DOI was a better predictor of recurrence than TFD, and they suggested a cut-off value of 4 mm for DOI. It was the first study suggesting a cut-off value for DOI [13]. One of the recent studies in 2015 by Ozbilen et al. investigated the effects of TFD and DOI as prognostic factors in endometrial cancer patients. They stated that while DOI was more valuable for nodal involvement, TFD was more valuable for adnexal involvement [10].

However, in all patient cohorts in these studies, all stages of endometrial cancer were included. Some of these patients were referred to adjuvant treatment.

Lymphadenectomy was not performed to all of them. All these factors could change survival and recurrence rates. To the best of our knowledge, this is the first study comparing TFD only in FIGO stage IB patients to evaluate recurrence and prognosis with a long-term follow-up which means comparing a more homogeneous group than other studies did.

One of the limitations of our study is its retrospective nature. Because only stage IB endometrial cancer patients were included, the recurrence and death rates were small, and this can affect the power of the study. However, at the same time, evaluating only stage IB patients makes the study stronger. In advanced stage, lots of risk factors could conflict the patients prognosis. Excluding other stages, we tried to minimize other negative prognostic factors and evaluate a more homogeneous group. Another limitation of the study is excluding nonendometrioid type endometrial cancer patients while including grade 3 endometrioid endometrial cancer patients. Nevertheless, adjuvant treatment of stage IB serous and clear cell endometrium cancer is chemotherapy as a first-line treatment thnn radiotherapy if indicated while radiotherapy is preferred in patients with grade 3 disease initially like grade 1 and 2 disease [18]. Large prospective series with more recurrence rate could show the prognostic importance of TFD in the future.

## 5. Conclusions

Our study showed that TFD does not affect the recurrence and survival in patients who underwent staging surgery, diagnosed as stage IB endometrioid endometrial cancer, and received adjuvant radiotherapy. Therefore, TFD is not an independent prognostic factor for patients with stage IB endometrioid endometrial cancer.

## Figures and Tables

**Table 1:** Risk groups of study populations according to the ESMO-ESGO-ESTRO Consensus Conference on Endometrial Cancer.

Risk group	*n* (%)
Intermediate	61 (45.8)
High-intermediate	42 (31.5%)
High	30 (22.5%)

ESMO: European Society for Medical Oncology; ESGO: European Society of Gynaecological Oncology; ESTRO: European Society for Radiotherapy and Oncology.

**Table 2:** Surgicopathologic characteristics of patients.

	Mean (range)
Age at diagnosis	61.5 years (40–85)
MT	20 mm (8–40)
DOI	14 mm (5–32)
TFD	6 mm (1–17)
TD	35 mm (15–65)
%MI	70% (50–95)
Follow-up time	95.1 months (10–214)
Recurrence time	37.6 months (9–84)
	*n* (%)
FIGO grade	
1	6 (4.5%)
2	97 (72.9%)
3	30 (22.6%)
Cytology	
Positive	1 (0.8%)
Negative	132 (99.2%)
LVSI	
Present	66 (49.6%)
Absent	67 (50.4%)
Recurrence	
Yes	9 (6.8%)
No	124 (93.2%)
Death	
Yes	2 (1.5%)
No	131 (98.5)

MT: myometrial thickness; DOI: depth of invasion; TFD: tumor-free distance; TD: tumor diameter; %MI: percentage of myometrial invasion; LVSI: lymphovascular space invasion; FIGO: International Federation of Gynecology and Obstetrics.

**Table 3:** Prediction of disease recurrence in the univariate model.

Covariate	OR (95% CI)	*p* value
TDF	0.934 (0.145–6.033)	0.943
DOI	1.031 (0.299–3.563)	0.961
%MI	1.006 (0.956–1.058)	0.823

DOI: depth of invasion; TFD: tumor-free distance; %MI: percentage of myometrial invasion; OR: odds ratio; CI: confidence interval.

**Table 4:** Prediction of disease recurrence in the multivariate model.

Covariate	OR (95% CI)	*p* value
TDF	2.85 (0.016–508.732)	0.691
DOI	0.612 (0.044–8.55)	0.715
%MI	1.039 (0.882–1.224)	0.65

DOI: depth of invasion; TFD: tumor-free distance; %MI: percentage of myometrial invasion; OR: odds ratio; CI: confidence interval.

## Data Availability

The retrospective data used to support the findings of this study are available from the corresponding author upon request.
